# Risk assessment of gene variants for neonatal hyperbilirubinemia in Taiwan

**DOI:** 10.1186/s12887-016-0685-8

**Published:** 2016-08-24

**Authors:** Yi-Hao Weng, Ya-Wen Chiu, Shao-Wen Cheng, Chun-Yuh Yang

**Affiliations:** 1 Division of Neonatology, Department of Pediatrics, Chang Gung Memorial Hospital, Chang Gung University College of Medicine, 199 Dunhua North Road, Taipei, 105 Taiwan; 2 Master Program in Global Health and Development, College of Public Health and Nutrition, Taipei Medical University, Taipei, Taiwan; 3 Department of Public Health, Kaohsiung Medical University, Kaohsiung, Taiwan

**Keywords:** Neonatal hyperbilirubinemia, Heme oxygenase-1, UDP-glucuronosyltransferase 1A1, Thalassemia, Hepatic solute carrier organic anion transporter 1B1

## Abstract

**Background:**

Hyperbilirubinemia is a common disorder during neonatal period in Taiwan. Gene variants may play an important role in the development of neonatal hyperbilirubinemia. The current study investigated the association between neonatal hyperbilirubinemia and common gene variants involving the production and metabolism of bilirubin.

**Methods:**

This prospective study enrolled 444 healthy infants born in the Chang Gung Memorial Hospital at Taipei from 2013–2015. Hyperbilirubinemia was defined as a total bilirubin ≥ 15 mg/dL. A log-binomial model was used to assess the risk of gene variants.

**Results:**

The most common genetic variant was short heme oxygenase (HO)-1 promoter GT-allele (<24 repeats) (39.4 %), followed by GA at nt388 in hepatic solute carrier organic anion transporter 1B1 (SLCO1B1) (31.1 %), GA at nt211 in UDP-glucuronosyltransferase 1A1 (UGT1A1) (29.3 %), ABO incompatibility (16.2 %), alpha thalassemia (5.0 %), and G6PD deficiency (3.2 %). The log-binomial analysis demonstrated greater risks of hyperbilirubinemia in infants with GA at nt211 in UGT1A1 (RR = 1.548; 95 % CI = 1.096–2.187), short HO-1 promoter GT-repeat (RR = 2.185; 95 % CI = 1.527–3.125), and G6PD deficiency (RR = 1.985; 95 % CI = 1.010–3.901). The other gene variants – including blood type, alpha thalassemia, and SLCO1B1 – carried no significant risk.

**Conclusions:**

G6PD deficiency, short HO-1 promoter GT-repeat and GA at nt211 in UGT1A1 are risk factors of neonatal hyperbilirubinemia. The data provide clinical evidence to explain the high incidence of neonatal hyperbilirubinemia in Taiwan.

## Background

Hyperbilirubinemia is a common disorder during neonatal period. It is associated with a variety of physiologic and pathologic conditions [1]. Common factors in relation to neonatal hyperbilirubinemia are breast milk feeding, isoimmune hemolytic disease and G6PD deficiency [2–5]. In addition, other gene variants – including heme oxygenase (HO)-1, hepatic solute carrier organic anion transporter 1B1 (SLCO1B1), and UDP-glucuronosyltransferase 1A1 (UGT1A1)—have been reported as risk factors of neonatal hyperbilirubinemia [6–9].

Asian infants are at greater risk of hyperbilirubinemia [10]. Gene variants may play an important role in the development of neonatal hyperbilirubinemia. In the current study, we examined 6 genetic variants involving the production and metabolism of bilirubin to verify their correlation with neonatal hyperbilirubinemia. Our data will provide clinical evidence to explain the high incidence of neonatal hyperbilirubinemia in Taiwan.

## Methods

### Study design

Healthy infants born in the Chang Gung Memorial Hospital at Taipei between January 2013 and August 2015 were eligible for enrollment. Infants with gestational age less than 35 weeks and birth weight less than 2000 g were not enrolled since they carry great risk for neonatal hyperbilirubinemia. In addition, ill neonates admitted to neonatal intensive care unit (NICU) were excluded. The Institutional Review Board of Chang Gung Memorial Hospital approved the study protocol. Informed consent was obtained from parents of enrolled infants.

### Clinical measures

Infants were routinely observed visually for the development of significant clinical jaundice. Bilirubin level was screened daily in all infants with non-invasive transcutaneous bilirubinmeter BiliCheck device (Spectrx Inc, Norcross, GA, USA) [11]. Serum bilirubin tests were performed with Unistat^TM^ bilirubinmeter (Cambridge Instruments, NY, USA). The protocol of phototherapy was modified from the guideline of 2004 American Academy of Pediatrics [12], as indicated for preterm infants < 37 weeks of gestation (≥7 mg/dL at < 24 h old, ≥ 9 mg/dL at 24–35 h old, ≥ 10 mg/dL at 36–47 h old, ≥ 12 mg/dL at 48–59 h old, ≥ 13 mg/dL at 60–71 h old, ≥ 14 mg/dL at 72–95 h old, and ≥ 15 mg/dL at ≥ 96 h old) and term infants (≥9 mg/dL at < 24 h old, ≥ 11 mg/dL at 24–35 h old, ≥ 12 mg/dL at 36–47 h old, ≥ 14 mg/dL at 48–59 h old, ≥ 15 mg/dL at 60–71 h old, ≥ 16 mg/dL at 72–95 h old, and ≥ 17 mg/dL at ≥ 96 h old). Hyperbilirubinemia was defined as total bilirubin ≥ 15 mg/dL during the hospital course [8].

Demographic data — including gender, delivery mode, birth weight and gestational age—were collected from birth records. The feeding type was classified into three categories: (1) formula feeding; (2) combination feeding of breast milk and formula, defined as at least one meal of breast milk and formula daily; (3) breast milk feeding.

### Laboratory measures

This study examined the following genes – including G6PD, blood type (ABO and Rh), HO-1, UGT1A1, alpha thalassemia, and SLCO1B1. All infants were screened for G6PD deficiency on the second day of life with blood samples from heel stick as a routine part of Taiwan’s national newborn screening program. The quantitative test for G6PD activity of red blood cells was performed to confirm the diagnosis of G6PD deficiency in those who had positive results from screening [2]. Blood type (ABO and Rh) was examined using a commercially available kit (Immucor Gamma, Norcross, GA, USA).

Total genomic DNA was isolated from cord blood cells by using the Puregene DNA Isolation Kit (Qiagen, Minneapolis, MN). The polymerase chain reaction (PCR) mixture comprises 10 mM Tris (pH 9.0), 50 mM KCL, 200 mM dNTPs, 1.25 mM MgCl_2_, 10 pmol each of primers, 20 μl DNA and 1 U Taq DNA polymerase (Gibco BRL). PCR is carried out in an automated thermocycler with optimal conditions. Restriction fragment length polymorphism (RFLP) and sequencing from products of PCR were conducted as means of detecting the known gene variants [8].

The HO-1 promoter gene containing GT repeats was amplified by PCR with a sense primer (5’-AGA GCC TGC AGC TTC TCA GA-3’) and an antisense primer (5’-ACA AAG TCT GGC CAT AGG AC-3’) according to a published protocol [13]. PCR products were analyzed in an automated DNA sequencer (ABI Prism 377, Foster City, CA). Sizes of GT repeats were calculated using GeneScan Analysis software (PE Applied Biosystems, Foster City, CA). To detect the alpha thalassemia-1 of Southeast Asia type, PCR was performed using the following primers – (a) 5` - GCG ATC TGG GCT CTG TGT TCT - 3`; (b) 5` - GTT CCC TGA GCC CCG ACA CG - 3`; (c) 5` - ACT GCA GCC TTG AAC TCC TG - 3` [14]. In addition, SLCO1B1 gene containing nt388 variant was amplified by PCR with a sense primer (5’-ATA ATG GTG CAA ATA AAG GGG-3’) and an antisense primer (5’-ACT ATC TCA AGG TGA TGC TCT A-3’) [15]. Furthermore, UGT1A1 gene containing nt211 variant was amplified by PCR with a sense primer (5’-CTC TAA GCA CAT CCC CAA GTA-3’) and an antisense primer (5’-TAA GCA AGT TTC CAT CCT TCA-3’) [15].

### Statistical analyses

The statistics were compiled using a commercially available program (SPSS 19.0 for Windows, SPSS Inc., Chicago, Illinois, USA). Categorical variables were analyzed using the chi-square test. For comparison between groups with quantitative variables, the null hypothesis that there was no difference between each group was tested by a one‐way analysis of variance (ANOVA). A log-binomial model (generalized linear model with a log link and a binomial distribution for the error term) was used to estimate the risk of neonatal hyperbilirubinemia in relation to gene variants after adjusting for possible confounders of neonatal factors – including feeding type, delivery mode, and gestational age. Relative risk with 95 % confidence intervals (CI) was expressed after adjusting for the control variables. Significance was defined as *p* < 0.05.

## Results

### Demographic information

Of the 520 parents approached for participation, 444 infants (85.4 %) were enrolled into this study. Their demographic data are listed in Table 1. The incidence of neonatal hyperbilirubinemia was 22.5 % (100/444). Only a few participants were preterm (3.6 %) and low birth weight infants (<2500 g) (0.9 %). Neonatal hyperbilirubinemia was noted in 100 infants (22.5 %). There was no significant difference in the gender, delivery mode, gestational age, birth weight, and feeding type between infants with and without hyperbilirubinemia.

**Table 1:** Demographic data of participants

Demographics	Hyperbilirubinemia		*p* value
Demographics	Yes	No	
	*N* = 100	*N* = 344	
Male sex	51 (51.0 %)	185 (53.8 %)	0.624
Cesarean section	27 (27.0 %)	112 (32.6 %)	0.291
Preterm	4 (4.0 %)	12 (3.5 %)	0.809
Birth weight (g)	3211 ± 330	3226 ± 372	0.715
Feeding type			0.814
Breast milk	52 (52.0 %)	192 (55.8 %)	
Formula	3 (3.0 %)	14 (4.1 %)	
Combination	45 (45.0 %)	138 (40.1 %)	

### Correlation of gene variants with neonatal hyperbilirubinemia

According to the classification of previous reports [16, 17], we divided the number of GT alleles into two categories: short (<24 repeats) and long (≥24 repeats) alleles. In total, the most common genetic variant was short HO-1 promoter GT-repeat (39.4 %), followed by GA at nt388 in SLCO1B1 (31.1 %), GA at nt211 in UGT1A1 (29.3 %), ABO incompatibility (defined as neonates with A or B blood type born to type O mothers [18]) (16.2 %), alpha thalassemia (5.0 %), G6PD deficiency (3.2 %), and Rh negative (0 %).

Table 2 illustrates the correlation of gene variants with neonatal hyperbilirubinemia. There was a significant correlation of neonatal hyperbilirubinemia with HO-1 promoter GT-repeat and nt211 in UGT1A1. Short HO-1 promoter GT-repeat and GA at nt211 in UGT1A1 were at greater risk of hyperbilirubinemia.

**Table 2:** Correlation of gene variants with neonatal hyperbilirubinemia by univariate analysis

Gene variant	Hyperbilirubinemia		*p* value
	Yes	No	
	*N* = 100	*N* = 344	
ABO incompatibility	17 (17.0 %)	55 (16.0 %)	0.809
G6PD deficiency	6 (6.0 %)	8 (2.3 %)	0.096
Alpha thalassemia	4 (4.0 %)	18 (5.2 %)	0.796
UGT1A1 (nt211)			0.015
GA	39 (39.0 %)	91 (26.5 %)	
GG	61 (61.0 %)	253 (73.5 %)	
SLCO1B1 (nt388)			0.449
GA	28 (28.0 %)	110 (32.0 %)	
GG	72 (72.0 %)	234 (68.0 %)	
HO-1 promoter (GT)n allele			
< 24 repeats (short)	59 (59.0 %)	116 (33.7 %)	<0.001
≥ 24 repeats (long)	41 (41.0 %)	228 (66.3 %)	

### Risk assessment by a log-binomial model

A log-binomial analysis to assess the risk of hyperbilirubinemia is shown in Table 3. There were greater risks of neonatal hyperbilirubinemia in infants with G6PD deficiency, short HO-1 promoter allele, and GA at nt211 in UGT1A1.

**Table 3:** Risk assessment of gene variants for neonatal hyperbilirubinemia by log-binomial analysis (*n* = 444)

Gene variant	Relative risk	95 % CI	*p* value
ABO incompatibility	1.075	0.685–1.687	0.754
G6PD deficiency	1.985	1.010–3.901	0.047
Alpha thalassemia	0.619	0.252–1.519	0.295
GA at nt211 in UGT1A1	1.548	1.096–2.187	0.013
GA at nt388 in SLCO1B1	0.921	0.627–1.353	0.675
Short HO-1 promoter GT-repeat	2.185	1.527–3.125	<0.001

## Discussion

The current study depicts the correlation of gene variants with neonatal hyperbilirubinemia in Taiwan. We used a log-binomial model to control the possible confounding factors. In addition, our study investigated six different genes involving the production and metabolism of bilirubin. Furthermore, we selected common gene variants in an attempt to explain the high incidence of neonatal hyperbilirubinemia in Taiwan. The data showed 3 gene variants – including G6PD deficiency, GA at nt211 in UGT1A1, and short HO-1 promoter GT-repeat – carried great risks for hyperbilirubinemia. To our knowledge, our study is the first prospective survey to determine the correlations of neonatal hyperbilirubinemia with HO-1 promoter GT-repeat and alpha-thalassemia in Taiwan.

HO is the rating-limiting enzyme to catalyze heme into bilirubin [19]. HO-1, one of HO isoforms, has been regarded as an inducible antioxidant [20]. The size of GT-repeat alleles in the HO-1 promoter can alter the inducibility of HO-1 [21]. Thus, a number of studies tried to verify the association between HO-1 promoter GT-repeat and neonatal jaundice. However, the results are still controversial [7, 16, 17, 22–25]. In our study, HO-1 promoter GT allele was relevant to neonatal hyperbilirubinemia. Our data demonstrated short HO-1 promoter GT-repeat is the most common gene variant. Thus, we speculate gene variants of HO-1 promoter GT allele contribute to the high prevalence of neonatal hyperbilirubinemia in the population of Taiwan.

UGT1A1 is an enzyme responsible for bilirubin conjugation. A missense mutation of G to A at nt211 in UGT1A1 is common [26]. Our study demonstrated that GA at nt211 in UGT1A1 is associated with neonatal hyperbilirubinemia, which is consistent with a number of previous reports [8, 26–28]. However, a correlation of GA at nt211 in UGT1A1 with neonatal hyperbilirubinemia was not noted in Caucasian population [27, 29]. Further study is needed to verify the difference between ethnicities.

It’s well documented that G6PD deficiency is a risk factor of neonatal hyperbilirubinemia [2, 3, 12]. In our study, the incidence of G6PD deficiency in infants with hyperbilirubinemia was 2.6-fold higher than that of infants without hyperbilirubinemia. However, the univariate analysis did not show a statistical difference. Nevertheless, the log-binomial analysis demonstrated a significant correlation of G6PD deficiency with neonatal hyperbilirubinemia after adjusting other confounding factors. In our study, infants with G6PD deficiency are at an increased risk for hyperbilirubinemia in the first few days of life even in the hospital free from agents that can potentially cause destruction of G6PD-deficient red cells. The data support our previous report in a large scale of population showing that neonatal hyperbilirubinemia in relation to G6PD deficiency is not associated with hemolysis [2].

SLCO1B1, also named as OATP2, is responsible for the transportation of unconjugated bilirubin. A relationship between nt388 in SLCO1B1 and neonatal hyperbilirubinemia has been documented [15, 30]. However, such correlation is in debate [8]. In this study, we did not find significant correlation of nt388 in SLCO1B1 with hyperbilirubinemia.

Our data indicate alpha thalassemia is not a risk factor of hyperbilirubinemia. The finding is similar to a retrospective report showing the incidence of hyperbilirubinemia was lower in the infants with alpha thalassemia [31]. We speculate that the destruction of red cells in infant with alpha thalassemia was decreased as a result of insufficient production of red cells. Therefore, their production of bilirubin was lower than infants without alpha thalassemia.

Blood group mismatch between a mother and newborn carries a substantial risk for neonatal hyperbilirubinemia [18]. However, we did not find blood group incompatibility as a significant factor of hyperbilirubinemia. We speculate three possible reasons. First, Rh incompatibility is very rare in Taiwan. Second, hemolytic anemia is not common in ABO incompatibility. Third, early phototherapy may reduce the development of hyperbilirubinemia. Therefore, we found a discrepancy of genetic influence between phototherapy and hyperbilirubinemia (data not shown). Taken together, we suggest that blood group incompatibility has only mild impact on the high incidence of neonatal hyperbilirubinemia in Taiwan.

A couple of methodological issues should be cautiously interpreted in this study. First, the sample size was relatively small in infants with hyperbilirubinemia. Nevertheless, the statistical power using the G∗Power 3.1 program to detect a significant association in HO-1 promoter allele was 99.1 %, which indicating sufficient power to achieve a low risk of type II error. Second, we enrolled infants whose parents were willing to participating in this study. One would question whether the gene variants in this design carry expected frequency. We believe the selection bias is minimal because of similar frequency with previous reports in Taiwan [2, 8, 13, 14, 18, 26].

## Conclusions

There are some critical findings in this study. We show G6PD deficiency, GA at nt211 in UGT1A1, and short HO-1 promoter GT-repeat possess great risks for neonatal hyperbilirubinemia. In contrast, the other 3 gene variants – including alpha thalassemia, blood group incompatibility, and SLCO1B1—are not related to hyperbilirubinemia. The findings suggest that a high incidence of neonatal hyperbilirubinemia in Taiwan may be derived from high prevalence of gene variants in G6PD, nt211 in UGT1A1, and HO-1 promoter GT-repeat. Overall, our data provide clinical implication of identifying infants at great risk of neonatal hyperbilirubinemia. Early intervention and close monitor are helpful in reducing severe hyperbilirubinemia for infants with G6PD deficiency, short HO-1 promoter GT-repeat, and GA at nt211 in UGT1A1.

## Abbreviation

HO-1: Heme oxygenase-1
nt: nucleotide
PCR: Polymerase chain reaction
SLCO1B1: Hepatic solute carrier organic anion transporter 1B1
UGT1A1: UDP-glucuronosyltransferase 1A1

## References

[1] Dennery PA, Seidman DS, Stevenson DK (2001). Neonatal hyperbilirubinemia. N Engl J Med.

[2] Weng YH, Chou YH, Lien RI (2003). Hyperbilirubinemia in healthy neonates with glucose-6-phosphate dehydrogenase deficiency. Early Hum Dev.

[3] Weng YH, Chiu YW (2010). Clinical characteristics of G6PD deficiency in infants with marked hyperbilirubinemia. J Pediatr Hematol Oncol.

[4] Gourley GR (2002). Breast-feeding, neonatal jaundice and kernicterus. Semin Neonatol.

[5] Cheng SW, Chiu YW, Weng YH (2012). Etiological analyses of marked neonatal hyperbilirubinemia in a single institution in Taiwan. Chang Gung Med J.

[6] Maruo Y, Morioka Y, Fujito H, Nakahara S, Yanagi T, Matsui K, Mori A, Sato H, Tukey RH, Takeuchi Y (2014). Bilirubin uridine diphosphate-glucuronosyltransferase variation is a genetic basis of breast milk jaundice. J Pediatr.

[7] Tiwari PK, Sethi A, Basu S, Raman R, Kumar A (2013). Heme oxygenase-1 gene variants and hyperbilirubinemia risk in North Indian newborns. Eur J Pediatr.

[8] Chang PF, Lin YC, Liu K, Yeh SJ, Ni YH (2013). Identifying term breast-fed infants at risk of significant hyperbilirubinemia. Pediatr Res.

[9] Chou HC, Chen MH, Yang HI, Su YN, Hsieh WS, Chen CY, Chen HL, Chang MH, Tsao PN (2011). 211G to a variation of UDP-glucuronosyl transferase 1A1 gene and neonatal breastfeeding jaundice. Pediatr Res.

[10] Setia S, Villaveces A, Dhillon P, Mueller BA (2002). Neonatal jaundice in Asian, white, and mixed-race infants. Arch Pediatr Adolesc Med.

[11] Weng YH, Chiu YW, Cheng SW (2012). Breast milk jaundice and maternal diet with Chinese herbal medicines. Evid Based Complement Alternat Med.

[12] American Academy of Pediatrics Subcommittee on H (2004). Management of hyperbilirubinemia in the newborn infant 35 or more weeks of gestation. Pediatrics.

[13] Chen YH, Lin SJ, Lin MW, Tsai HL, Kuo SS, Chen JW, Charng MJ, Wu TC, Chen LC, Ding YA (2002). Microsatellite polymorphism in promoter of heme oxygenase-1 gene is associated with susceptibility to coronary artery disease in type 2 diabetic patients. Hum Genet.

[14] Chang JG, Lee LS, Lin CP, Chen PH, Chen CP (1991). Rapid diagnosis of alpha-thalassemia-1 of southeast Asia type and hydrops fetalis by polymerase chain reaction. Blood.

[15] Huang MJ, Kua KE, Teng HC, Tang KS, Weng HW, Huang CS (2004). Risk factors for severe hyperbilirubinemia in neonates. Pediatr Res.

[16] Yang H, Wang Q, Zheng L, Lin M, Zheng XB, Lin F, Yang LY (2015). Multiple Genetic Modifiers of Bilirubin Metabolism Involvement in Significant Neonatal Hyperbilirubinemia in Patients of Chinese Descent. PLoS One.

[17] Bozkaya OG, Kumral A, Yesilirmak DC, Ulgenalp A, Duman N, Ercal D, Ozkan H (2010). Prolonged unconjugated hyperbilirubinaemia associated with the haem oxygenase-1 gene promoter polymorphism. Acta Paediatr.

[18] Weng YH, Chiu YW (2009). Spectrum and outcome analysis of marked neonatal hyperbilirubinemia with blood group incompatibility. Chang Gung Med J.

[19] Weng YH, Yang G, Weiss S, Dennery PA (2003). Interaction between heme oxygenase-1 and −2 proteins. Journal Biol Chem.

[20] Weng YH, Tatarov A, Bartos BP, Contag CH, Dennery PA (2000). HO-1 expression in type II pneumocytes after transpulmonary gene delivery. Am J Physiol Lung Cell Mol Physiol.

[21] Yamada N, Yamaya M, Okinaga S, Nakayama K, Sekizawa K, Shibahara S, Sasaki H (2000). Microsatellite polymorphism in the heme oxygenase-1 gene promoter is associated with susceptibility to emphysema. Am J Hum Genet.

[22] Zhou Y, Wang SN, Li H, Zha W, Peng Q, Li S, Chen Y, Jin L (2014). Quantitative trait analysis of polymorphisms in two bilirubin metabolism enzymes to physiologic bilirubin levels in Chinese newborns. J Pediatr.

[23] Kaplan M, Renbaum P, Hammerman C, Vreman HJ, Wong RJ, Stevenson DK (2014). Heme oxygenase-1 promoter polymorphisms and neonatal jaundice. Neonatology.

[24] Katayama Y, Yokota T, Zhao H, Wong RJ, Stevenson DK, Taniguchi-Ikeda M, Nakamura H, Iijima K, Morioka I (2015). Association of HMOX1 gene promoter polymorphisms with hyperbilirubinemia in the early neonatal period. Pediatr Int.

[25] Kanai M, Akaba K, Sasaki A, Sato M, Harano T, Shibahara S, Kurachi H, Yoshida T, Hayasaka K (2003). Neonatal hyperbilirubinemia in Japanese neonates: analysis of the heme oxygenase-1 gene and fetal hemoglobin composition in cord blood. Pediatr Res.

[26] Huang CS, Chang PF, Huang MJ, Chen ES, Hung KL, Tsou KI (2002). Relationship between bilirubin UDP-glucuronosyl transferase 1A1 gene and neonatal hyperbilirubinemia. Pediatr Res.

[27] Long J, Zhang S, Fang X, Luo Y, Liu J (2011). Neonatal hyperbilirubinemia and Gly71Arg mutation of UGT1A1 gene: a Chinese case–control study followed by systematic review of existing evidence. Acta Paediatr.

[28] Maruo Y, Nishizawa K, Sato H, Doida Y, Shimada M (1999). Association of neonatal hyperbilirubinemia with bilirubin UDP-glucuronosyltransferase polymorphism. Pediatrics.

[29] Hanchard NA, Skierka J, Weaver A, Karon BS, Matern D, Cook W, O'Kane DJ (2011). UGT1A1 sequence variants and bilirubin levels in early postnatal life: a quantitative approach. BMC Med Genet.

[30] Liu J, Long J, Zhang S, Fang X, Luo Y (2013). Polymorphic variants of SLCO1B1 in neonatal hyperbilirubinemia in China. Ital J Pediatr.

[31] Ko TM, Hwang WJ, Chen SH, Lee TY, Hsieh GY, Lee CY (1990). Alpha-thalassemia minor and neonatal hyperbilirubinemia. J Formos Med Assoc.

